# The Austrian biodatabase for chronic myelomonocytic leukemia (ABCMML): A representative and useful real-life data source for further biomedical research

**DOI:** 10.1007/s00508-019-1526-1

**Published:** 2019-07-18

**Authors:** Klaus Geissler, Eva Jäger, Agnes Barna, Michael Gurbisz, Renate Marschon, Temeida Graf, Elmir Graf, Bojana Borjan, Ruth Jilch, Christoph Geissler, Gregor Hoermann, Harald Esterbauer, Ilse Schwarzinger, Thomas Nösslinger, Michael Pfeilstöcker, Heinz Tüchler, Regina Reisner, Thamer Sliwa, Felix Keil, Peter Bettelheim, Sigrid Machherndl-Spandl, Bernhard Doleschal, Otto Zach, Ansgar Weltermann, Sonja Heibl, Josef Thaler, Armin Zebisch, Heinz Sill, Reinhard Stauder, Gerald Webersinke, Andreas Petzer, Rajko Kusec, Ernst Ulsperger, Bruno Schneeweiss, Jörg Berger, Leopold Öhler, Ulrich Germing, Wolfgang R. Sperr, Paul Knöbl, Ulrich Jäger, Peter Valent

**Affiliations:** 1 Sigmund Freud University, Vienna, Austria; 2 Department of Internal Medicine V with Hematology, Oncology and Palliative Medicine, Hospital Hietzing, Wolkersbergenstraße 1, 1130 Vienna, Austria; 3 Department of Laboratory Medicine, Medical University of Vienna, Vienna, Austria; 4 Blood Transfusion Service, Blood Transfusion Service for Upper Austria, Austrian Red Cross, Linz, Austria; 5 Department of Internal Medicine I with Hematology with Stem Cell Transplantation, Hemostaseology and Medical Oncology, Ordensklinikum Linz Barmherzige Schwestern – Elisabethinen, Linz, Austria; 6 Internal Medicine V with Hematology and Oncology, Medical University of Innsbruck, Innsbruck, Austria; 7 Department of Laboratory Medicine, Hospital Hietzing, Vienna, Austria; 8 Central Institute of Medical and Chemical Laboratory Diagnostics, Medical University of Innsbruck, Innsbruck, Austria; 9 Department of Internal Medicine III, Hanusch Hospital, Vienna, Austria; 10 Department of Internal Medicine IV, Hospital Wels-Grieskirchen, Wels, Austria; 11 Department of Internal Medicine, Division of Hematology, Medical University of Graz, Graz, Austria; 12 School of Medicine, University of Zagreb, University Hospital Dubrava, Zagreb, Croatia; 13 Department of Internal Medicine, Hospital Horn, Horn, Austria; 14 Department of Internal Medicine, Hospital Kirchdorf, Kirchdorf, Austria; 15 Department of Internal Medicine, Hospital Schwarzach, Schwarzach, Austria; 16 Department of Internal Medicine/Oncology, St. Josef Hospital, Vienna, Austria; 17 Department of Hematology, Oncology, and Clinical Immunology, Heinrich-Heine-University, Düsseldorf, Germany; 18 Department of Internal Medicine I, Division of Hematology and Hemostaseology, Medical University of Vienna, Vienna, Austria; 19 Ludwig Boltzmann Institute for Hematology and Oncology (LBI HO), Medical University of Vienna, Vienna, Austria

**Keywords:** CMML, NGS, CFU-GM, RAS, *In vitro* culture

## Abstract

In the Austrian biodatabase for chronic myelomonocytic leukemia (ABCMML) clinicolaboratory real-life data have been captured from 606 CMML patients from 14 different hospitals over the last 30 years. It is the only large biodatabase worldwide in which functional methods such as semisolid in vitro cultures complement modern molecular methods such as next generation sequencing. This provides the possibility to comprehensively study the biology of CMML. The aim of this study was to compare patient characteristics with published CMML cohorts and to validate established prognostic parameters in order to examine if this real-life database can serve as a representative and useful data source for further research. After exclusion of patients in transformation characteristics of 531 patients were compared with published CMML cohorts. Median values for age, leukocytes, hemoglobin, platelets, lactate dehydrogenase (LDH) and circulating blasts were within the ranges of reported CMML series. Established prognostic parameters including leukocytes, hemoglobin, blasts and adverse cytogenetics were able to discriminate patients with different outcome. Myeloproliferative (MP) as compared to myelodysplastic (MD)-CMML patients had higher values for circulating blasts, LDH, RAS-pathway mutations and for spontaneous myelomonocytic colony growth in vitro as well as more often splenomegaly. This study demonstrates that the patient cohort of the ABCMML shares clinicolaboratory characteristics with reported CMML cohorts from other countries and confirms phenotypic and genotypic differences between MP-CMML and MD-CMML. Therefore, results obtained from molecular and biological analyses using material from the national cohort will also be applicable to other CMML series and thus may have a more general significance.

## Introduction

Chronic myelomonocytic leukemia (CMML) is a hematopoietic malignancy of elderly people that is characterized by leukocytosis with monocytes and granulocytic cells in all stages of development, marked dysmyelopoiesis, a variable course, unresponsiveness to aggressive chemotherapy and an inherent risk of transformation to acute myeloid leukemia (AML) [1, 2]. With respect to the presence of myeloproliferation CMML was originally subdivided into myeloproliferative disorder (MPD)-CMML (white blood cell [WBC] count >13 × 10^9^/L) versus myelodysplastic syndrome (MDS)-CMML (WBC count ≤13 × 10^9^/L) by the French-American-British group (FAB) criteria [3]. Since CMML is characterized by features of both MDS and myeloproliferative neoplasm (MPN) the World Health Organization (WHO) classification of 2000 assigned CMML to the mixed category MDS/MPN [4]. Chronic myelomonocytic leukemia was further subclassified into CMML-1 (<5% circulating blasts and <10% bone marrow, BM blasts) and CMML-2 (5–19% circulating blasts, 10–19% BM blasts). In the 2008 WHO classification it was recommended that cases of CMML with eosinophilia should be investigated for a *PDGFRB *gene abnormality and if detected, the case should be classified as a myeloid neoplasm with eosinophilia associated with *PDGFRB *rearrangement [1]. Recent evidence has shown that an even more precise prognostication can be obtained with 3 blast-based groupings: CMML-0, a category for cases with <2% blasts in peripheral blood (PB) and <5% blasts in bone marrow, CMML-1 for cases with 2–4% blasts in PB and/or 5–9% blasts in BM, and CMML-2 for cases with 5–19% blasts in PB, 10–19% in BM, and/or when any Auer rods are present. Therefore, the revision 2016 incorporates the CMML-0 category into the classification scheme [5].

In a large series of 1832 patients captured in the international CMML database that merged CMML registries from 8 tertiary cancer centers across 3 different countries between July 1981 and June 2014 the median age at diagnosis was 70 years (range 16–93 years), with a male predominance (67%) [6]. Most patients were evenly subcategorized as MPN-CMML (49.8%) versus MDS-CMML (50.2%). Splenomegaly was demonstrable in 25% of all cases. The median overall survival of CMML patients is approximately 30 months, with one third evolving to AML while the others die from the consequences of cytopenia or comorbidities. Several CMML cohorts have been reported and prognostic models including hematologic, cytogenetic and molecular data have been developed to stratify patients into groups that are predictive for overall survival but there is no single universally used score [7–12]. Allogeneic transplantation, which is the only curative therapy, is rarely feasible because of age and/or comorbidities. In patients ineligible for transplantation, intensive chemotherapy results in low response rates and short response duration [13]. Hydroxyurea is used to control myeloproliferation [14]. The cytidine analogues azacytidine (AZA) and decitabine (5-aza-2′-deoxycytidine) have demonstrated some efficacy in delaying disease progression in advanced CMML and were approved for the treatment of CMML [15–17].

In the ABCMML clinicolaboratory real-life data have been collected from CMML patients from different centers over the last 30 years. It is the only large biodatabase worldwide in which functional methods such as semisolid in vitro cultures complement modern molecular methods such as next generation sequencing providing the possibility to comprehensively study and better understand the pathophysiology of CMML. The aim of this study was to compare patient characteristics with published CMML cohorts and to validate established prognostic parameters in order to examine if this real-life database may serve as a representative and useful data source for further research.

## Patients and methods

Between 1988 and 2018, 606 patients with CMML from 14 hospitals were captured in the ABCMML. This database retrospectively collected epidemiologic, hematologic, biochemical, clinical, immunophenotypic, cytogenetic, molecular and biologic data of patients with CMML from different centers in a real-life setting. Internal review board approval was obtained at each institution. Clinical and laboratory routine parameters were obtained from patient records. A detailed central manual retrospective chart review was carried out to ensure data quality before analysis of data from institutions. Data curation included the extraction of discrete data elements from patient records, a check for accuracy and consistency of data, and a verification that baseline data were reflective of CMML that was strictly defined according to WHO criteria. Since the necessary information was not available in all patient records it was not possible to reclassify all cases according to the most recent WHO classification; however, patients with a history of antecedent CMML and 20% or more blasts in PB and/or BM were uniformly considered as CMML-derived secondary AML.

In one of the centers (Medical University of Vienna) the assessment of hematopoietic colony formation in vitro has been an integral part of the diagnostic work-up in patients with suspected myeloid malignancies for many years [18]. Samples of PB and/or BM were taken after written informed consent was provided from patients. Cytogenetic analysis was performed using G‑banding according to standard techniques on BM cells 24–48 h in unstimulated culture. Chromosome aberrations were classified according to the International System for Human Cytogenetic Nomenclature (ISCN). The CMML-specific cytogenetic risk classification was low for normal karyotype and isolated-Y, intermediate for other abnormalities and high for trisomy 8, complex karyotype (≥3 abnormalities), and abnormalities of chromosome 7 [8]. In general samples were taken before any disease-modifying treatment (i.e. allogeneic stem cell transplantation, aggressive chemotherapy or hypomethylating agents).

### Semisolid in vitro cultures

Colony-forming unit granulocyte-macrophage (CFU-GM) growth was assessed in semisolid cultures without growth factors as previously described in one central laboratory [19]. Mononuclear cells (MNC) were isolated from PB of patients by Ficoll-Hypaque density gradient centrifugation (density, 1.007 g/mL, 400 g for 40 minutes). The low-density cells were collected from the interface between density solution and plasma, washed twice, and resuspended in Iscove’s modified Dulbecco’s medium (GIBCO, Paisley, Scotland). In unstimulated cultures PBMNCs were cultured in 0.9% methylcellulose, 30% fetal calf serum (FCS; INLIFE, Wiener Neudorf, Austria), 10% bovine serum albumin (Behring, Marburg, Germany), α‑thioglycerol (10^–4^ mol/L) and Iscove’s modified Dulbecco’s medium. Cultures were plated in duplicates or triplicates at 25–100 × 10^3^ PBMNC/mL. In some cases the numbers of MNC chosen in the experiments were based on the colony growth in prior cell cultures in the respective patient in order to optimize evaluation of CFU-GM formation. Plates were incubated at 37 °C, 5% CO_2_, and full humidity. After a culture period of 14 days, cultures were examined under an inverted microscope. Aggregates with more than 40 translucent, dispersed cells were counted as CFU-GM. The CFU-GM data are expressed as mean values from cultures.

### Molecular analysis

Molecular analyses were performed by three laboratories using next-generation (NG) sequencing with amplicon-based target enrichment. Genomic DNA was isolated from MNC fractions of PB or BM samples according to standard procedures. The following genes (exon number) were analyzed:

Lab 1:* ASXL1 *(13),* ATRX* (1-35),* BCOR *(3-15),* BRAF *(15)*, CBL *(8-9)*, CDKN2A *(1-3)*, CEBPA *(1),* CSF1R *(3-21),* CSF3R *(3-17),* DNMT3A *(2-23),* EGFR *(18-20),* ETV6 *(1-8),* EZH2 *(2-20),* FLT3 *(10, 16, 20),* GNAS *(1-9, 13),* IDH1 *(4),* IDH2 *(4),* JAK2 *(12, 14),* KDM6A *(1-11, 13-28),* KIT *(1, 3‑21),* KRAS *(2-3), *MET *(2-21),* NF1 *(1-58),* NPM1 *(11),* NRAS *(2-3),* PRP40B *(2-6, 8‑17, 19-25),* PTPN11 *(2-25),* RUNX1 *(3-8),* SETBP1 *(2-6),* SF1 *(2-12),* SF3A1 *(1-15),* SF3B1 *(7-17),* SRSF2 *(1),* STAG2 *(4-33),* TET2 *(3-11),* TP53 *(2-10),* U2AF1 *(2-8),* WT1 *(1-10),* ZRSR2 *(1-11)

Lab 2:* ABL1 *(4-9),* ASXL1 *(9, 11-12),* BRAF *(15),* CALR *(9),* CBL *(8-9),* CEBPA *(all),* CSF3R *(all),* DNMT3A *(all),* ETV6 *(all),* EZH2 *(all),* FLT3 *(13-15, 20),* HRAS *(2-3),* IDH1 *(4),* IDH2 *(4),* JAK2 *(all),* KIT *(2, 8‑11, 13, 17-18),* KRAS *(2-3),* MPL *(10),* NPM1 *(10-11),* NRAS *(2-3),* PTPN11 *(3, 7‑13),* RUNX1 *(all),* SETBP1 *(4),* SF3B1 *(10-16),* SRSF2 *(1),* TET2 *(all),* TP53 *(all),* U2AF1 *(2, 6),* WT1 *(5-9),* ZRSR2 *(all)

Lab 3:* ABL1 *(4-9),* ASXL1 *(9, 11-12),* BCOR *(2-15),* BIRC3 *(6-9),* BRAF *(15),* BTK *(15), *CALR *(9),* CBL *(8-9, 12),* CEBPA *(all),* CSF3R *(all),* DNMT3A *(all),* ETV6 *(all),* EZH2 *(all),* FLT3 *(13-15, 20),* HRAS *(2-4),* IDH1 *(4),* IDH2 *(4),* JAK2 *(all),* KIT *(2, 8‑11, 13, 17-18),* KRAS *(2-4),* MPL *(10),* MYD88 *(5),* NF1 *(1-58),* NOTCH *(3′UTR),* NPM1 *(10-11),* NRAS *(2-4),* PLGC2 *(19-20, 24),* PTPN11 *(3, 7‑13),* RUNX1 *(all),* SETBP1 *(4),* SF3B1 *(10-16),* SRSF2 *(1),* STAG2 *(3-35),* TET2 *(all),* TP53 *(all),* U2AF1 *(2, 6),* WT1 *(6-10, 26-28, 34),* ZRSR2 *(all)

After sequencing on an Illumina MiSeq platform (Illumina Inc, San Diego, CA, USA), data were demultiplexed and reads were aligned against the human reference genome hg19. Variant annotation and interpretation were performed using the following databases and software tools:

- Lab 1: ClinVar and dsSNP-NCBI (*N*ational *C*enter for *B*iotechnology *I*nformation), COSMIC (*C*atalogue *o*f *S*omatic *M*utations *i*n *C*ancer), EGB (Ensembl Genomes Browser), ESP (Exome Sequencing Project) g1000 (1000 genomes), cg69, PolyPhen (*Poly*morphism *Phen*otyping), SIFT (*S*orting *I*ntolerant *f*rom *T*olerant), LRT and MutationTaster.
- Lab 2: SOPHiA DDM (Sophia Genetics Inc, Saint Sulpice, Switzerland), ExAC (Exome Aggregation Consortium), G1000 (1000 genomes), ESP (Exome Sequencing Project), COSMIC (*C*atalogue *o*f *S*omatic *M*utations *i*n *C*ancer), ClinVar, dbSNP, CG69, dbNSFP (database of human nonsynonymous SNPs and their functional predictions), GnomAD (Genome Aggregation Database)
- Lab 3: ClinVar and dsSNP-NCBI (*N*ational *C*enter for *B*iotechnology *I*nformation), COSMIC (*C*atalogue *o*f *S*omatic *M*utations *i*n *C*ancer), ExAC (Exome Aggregation Consortium), gnomAD (Genome Aggregation Database), TOPMed, IARC TP53, Align-GVGD, SIFT (*S*orting *I*ntolerant *f*rom *T*olerant) and MutationTaster.

In case of conflicting results for the pathogenicity of a variant, the underlying data were manually rechecked. Variants were considered (likely) benign unless they satisfied all of the following conditions: the mutation occurred in a protein coding region, the mutation function was not synonymous, the annotation from ClinVar was not benign, and the change was not found at a frequency of 1% or higher in a population. Clearly pathogenic variants and variants of unknown significance were retained as potential mutations. A VAF of 5% or higher was considered as positive for analysis.

### Statistical analysis

Statistical analyses were performed with the SPSS version 19.0.0 (SPSS Inc). The log-rank test was used to determine if individual parameters were associated with overall survival (OS), which was defined as the time from sampling to death or last follow-up and data from all samples which were available were used for the analysis. Dichotomous variables were compared between different groups with the use of the χ^2^-test. The Mann-Whitney U‑test was used to compare unmatched groups when continuous variables were not normally distributed. Results were considered significant at *P* < 0.05.

## Results

**Fig. 1:**
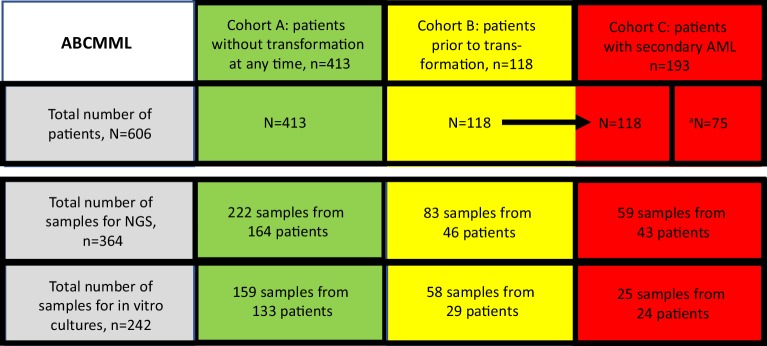
Summary of patients and samples collected in the Austrian biodatabase for chronic myelomonocytic leukemia (ABCMML). The discrepancy between the number of samples and patients is due to the fact that serial samples were taken in a proportion of patients. ^a^Patients with transformation before inclusion into the biodatabase. *NGS* next generation sequencing, *AML* acute myeloid leukemia

Data from 606 patients with CMML in different stages of development were used for this study (Fig. 1). There were 413 patients without transformation at any time and 118 patients who were included in the study before transformation but later developed secondary AML during follow-up. The study also included 75 patients who had CMML-derived AML but had already had transformation before entering the study. Thus, 193 patients with secondary AML were available for analysis. A total of 364 samples were available for molecular analysis with NGS and a total of 242 samples for in vitro studies. In order to compare the clinicohematological data of the ABCMML cohort with published CMML series patients with secondary AML were excluded for this analysis. The clinical and laboratory data of the 531 CMML patients of the ABCMML and of other reported CMML cohorts are shown in Table 1. The median age of patients in the cohort was 72 years being in the range of other cohorts (65–74 years). As seen in other CMML series there was also a male predominance (63%) in the ABCMML patients. In general, all median values of the cohort including leukocytes, hemoglobin, platelets, LDH and PB blast cells were within the ranges of CMML cohorts reported from other groups. The median OS which was highly variable between cohorts (12–52 months) was 29 months in the ABCMML.

**Table 1:** Characteristics of CMML patients captured in the ABCMML and patients from other published CMML cohorts

Cohort	ABCMML *N* = 531	Texas [7] *N* = 213	France [8] *N* = 312	Spain [9] *N* = 558	Düsseldorf [9] *N* = 274	Pavia [11] *N* = 214	Munich [11] *N* = 260	Mayo [12] *N* = 324
Age in years; median (range)	72 (34–93)	65 (20–88)	74 (41–93)	73 (19–99)	70 (41–98)	72 (28–99)	74 (0.2–155)	71 (18–95)
Sex (male); *n* (%)	337 (63)	150 (70)	210 (67)	377 (68)	191 (70)	151 (71)	190 (73)	216 (67)
WBC × 10^9^/L; median (range)	11.9 (1.6–271)	20.4 (2.1–352)	12.4 (2.0–367)	10 (1–156)	13 (1–150)	9.8 (1.2–126)	11.0 (2.0–87.0)	12.3 (1.8–264.8)
Hb g/dL; median (range)	10.9 (4.3–16.5)	10.2 (5.2–15.6)	11.3 (5.3–16.9)	11 (1–19)	11 (2–17)	11.6 (6–16.6)	11.5 (5.9–17)	10.8 (6.4–16.9)
PLT × 10^9^/L; Median (range)	109 (1–1181)	87 (4–706)	120 (9–1098)	123 (4–928)	105 (1–979)	124 (4–943)	103 (3–1385)	102 (10–840)
PB blasts %; Median (range)	0 (0–19)	0 (0–22)	NA	NA	NA	NA	NA	0 (0–19)
BM blasts %; Median (range)	NA	4 (0–19)	NA	3 (0–19)	6 (0–19)	3 (1–18)	6 (0–19)	3 (0–19)
LDH U/L; Median (range)	251 (67–3380)	783 (270–5310)	NA	394 (104–2976)	277 (97–7743)	NA	NA	226 (84–1296)
Intermediate + high risk karyotype^a^; *n* (%)	81/278 (29)	70/205 (34)	60/295 (20)	103/532 (19)	86/232 (37)	40/190 (21)	42/260 (16)	79/317 (25)
Median survival (months)	29	12	32	31	25	48	51	29
Molecular analysis by NGS	Yes	No	Yes	Yes	Yes	Yes	Yes	Yes
Functional analysis by in vitro cultures	Yes	No	No	No	No	No	No	No

^a^The cytogenetic risk classification was performed as described in Materials and Methods.

*WBC* white blood cells, *Hb* hemoglobin, *PLT* platelets, *PB* peripheral blood, *BM* bone marrow, *LDH* lactate dehydrogenase, *NGS* next-generation sequencing, *NA* not available

**Fig. 2:**
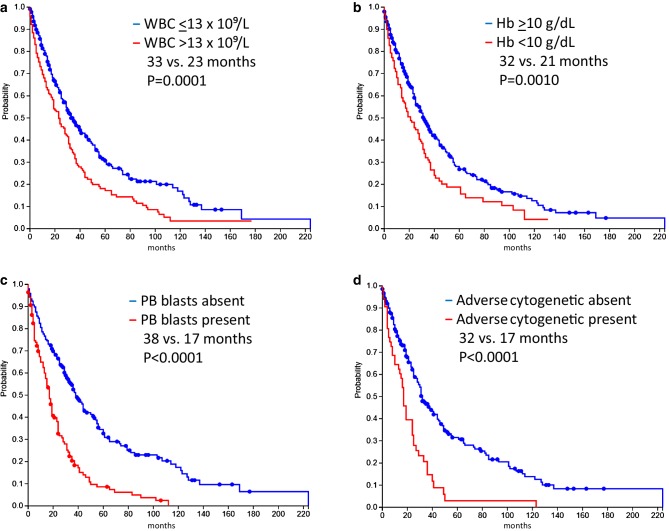
Kaplan-Meier survival analysis and median overall survival of four established prognostic variables such as leukocytes (**a**), haemoglobin (**b**), peripheral blood blasts (**c**) and adverse cytogenetic features (**d**). The cytogenetic risk classification was performed as described in Materials and Methods. *WBC* White blood cells, *Hb* hemoglobin, *PB* peripheral blood

Since there is no universally accepted prognostic score in patients with CMML different hospitals may use different scores. Therefore, published scores cannot be easily validated in a real-life data collection since parameters for the respective scores are different and may be missing in a significant number of patients. Therefore, it was deemed more appropriate to use single established prognostic parameters which were available in the majority of the patients to characterize the patient cohort in comparison to other cohorts. As shown in Fig. 2a–d established prognostic parameters including leukocyte count ≤13 vs. >13 × 10^9^/L, hemoglobin level ≥10 vs. <10 g/dL, the absence vs. presence of blasts in PB and the absence vs. presence of adverse cytogenetics were able to discriminate patients with different outcome.

**Fig. 3:**
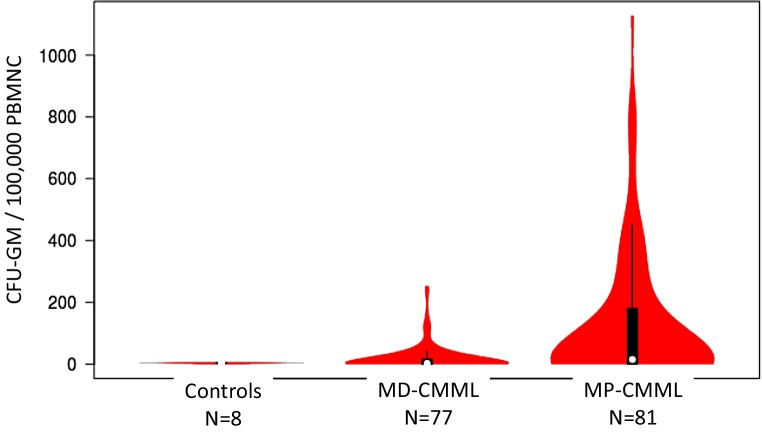
Violin plot of growth factor independent myeloid colony growth in patients with MD-CMML and MP-CMML. Cultures were performed as described in Materials and Methods. The number of CFU-GM per 10^5^ PBMNC is given

The MPN phenotype of CMML has previously been shown to be associated with leukocytosis, monocytosis, hepatomegaly, splenomegaly, constitutional symptoms and RAS mutations [20, 21]. Therefore, it was found to be important to do such a comparison in the ABCMML. As shown in Table 2 MP-CMML (WBC >13 × 10^9^/L) as compared to MD-CMML (≤13 × 10^9^/L) had higher values for PB blasts, LDH, and RAS-pathway mutations (including mutations in *NRAS, KRAS, CBL, NF1,* and *PTPN11*) as well as more often splenomegaly. Moreover, as shown in Table 2 and Fig. 3, a higher spontaneous myeloid colony growth in vitro was found in patients with MP-CMML as compared to MD-CMML which has not previously been published by other groups.

**Table 2:** Clinical and laboratory features in patients with MP-CMML and MD-CMML

Variables	All CMML patients (*n* = 531)	MP-CMML patients (*n* = 243)	MD-CMML patients (*n* = 288)	*P*-value
Age in years; median (range)	72 (34–93)	73 (34–92)	72 (36–93)	0.9282
Sex (male); *n* (%)	337 (63)	149 (61)	188 (65)	0.3445
WBC × 10^9^/L; median (range)	11.9 (1.6–271)	25.2 (13.1–271)	6.45 (1.6–12.9)	<0.0001
Hemoglobin g/dL, median (range)	10.9 (4.3–16.5)	10.9 (5.2–16.1)	11 (4.3–16.5)	0.8572
Platelets × 10^9^/L; median (range)	109 (1–1181)	103 (3–1148)	115 (2–1181)	0.6312
PB blasts %; median (range)	0 (0–19)	0 (0–18)	0 (0–19)	<0.0001
LDH; U/L; median (range)	251 (67–3380)	300 (67–1958)	210 (102–3380)	<0.0001
Splenomegaly; *n* (%)	87/241 (36)	56/127 (44)	31/114 (27)	0.0064
Median survival (months)	29	23	33	0.0001
Patients with mutations in RASopathy genes ≥5% VAF; *n* (%)	92/209 (44)	59/110 (54)	33/99 (33)	0.0032
Patients with spontaneous CFU-GM growth >20/100,000 PBMNC; *n* (%)	52/158 (33)	35/81 (43)	17/77 (22)	0.0047

CMML was subdivided into myeloproliferative CMML (MP-CMML; WBC count >13 × 10^9^/L) versus myelodysplastic CMML (MD-CMML; WBC count ≤13 × 10^9^/L) according to the FAB criteria [3]

## Discussion

The CMML is a rare disease of elderly people with a high unmet medical need due to the fact that the disease may severely impact the quality of life of patients, shorten survival and current treatment options are unsatisfactory. A better understanding of the pathophysiology may provide a chance to improve treatment outcome. In the past, cooperative multicenter studies of this entity have been very difficult and any information derived from patients with CMML managed in a real-life situation is welcome and may have the potential to increase knowledge about this disease. Therefore, some years ago the ABCMML was initiated which collects epidemiologic, hematologic, biochemical, clinical, cytogenetic, molecular and biologic data elements from different centers in a real-life setting. The fact that the biodatabase can provide information derived from molecular as well as from functional studies fuels hope to get a more comprehensive view and deeper insight into the complex pathophysiology of this hematologic malignancy than by either method alone. In order to see if this real-life database may serve as a representative and useful data source for further research, patient characteristics were first compared with published CMML cohorts and established prognostic parameters had to be validated.

Some interesting results from this database have already been reported. In particular in vitro studies have been a main focus of research for many years and distinguish the ABCMML from all other published CMML cohorts. This CMML project originally started with the observation that in vitro formation of CFU-GM without exogenous growth factors is a recurrent finding in a subset of patients with CMML [22]. Subsequently, it was found that spontaneous myeloid colony formation in CMML depends on the presence of endogenously produced granulocyte/macrophage colony-stimulating factor (GM-CSF) [19]. Moreover, it was reported that CMML patients with high spontaneous CFU-GM growth (>100/10^5^ PBMNC) have a worse prognosis compared with patients with low CFU-GM growth [23]. Later GM-CSF hypersensitivity has been reported as a feature of myeloid progenitors in CMML [24]. Moreover, molecular alterations of these components in murine hematopoietic cells can lead to a CMML-like disease in vivo and to spontaneous myeloid colony formation in vitro due to hypersensitivity of granulomonocytic precursors against GM-CSF [25–28]. Finally, a close correlation of RAS pathway mutations and high spontaneous CFU-GM growth in patients with CMML was demonstrated [29] strongly suggesting that the spontaneous in vitro growth of myeloid colonies (CFU-GM) may be a useful functional parameter of RAS pathway activation. In this study it could be shown that the MPN phenotype of CMML is associated with an increased spontaneous formation of CFU-GM suggesting that the development of myeloproliferation is accompanied by a successive loss of growth factor dependence of clonal cells.

The use of real-world data is associated with some limitations that have to be considered. One has to be aware that in a real-life collection such as the ABCMML not all data are available which one would like to have and which are predefined in a systematic prospective study. Multivariate analyses, therefore, are difficult to perform with an incomplete real-life dataset. Due to the fact that there are a number of different published prognostic scores in patients with CMML but no single universally accepted score, different hospitals often apply different scores. Therefore, all these published scores cannot be easily validated in a real-life data collection. Since single established prognostic parameters were available in the majority of the patients, it was found to be more appropriate to use these parameters to characterize the patient cohort in comparison to other cohorts. Moreover, the classification of CMML subcategories has changed over the last 30 years and reclassification based on available data and according to the most recent WHO classification could not be performed in every patient from previous years. In a retrospective study, however, it is possible and probably useful to categorize patients according to the fact if patients have developed secondary AML during observation. Therefore, the patients are grouped into one of three categories: patients without evidence of AML development at any time (CMML without transformation, cohort A), patients who developed AML during follow up (CMML pretransformation, cohort B) and patients after transformation to sAML (CMML-derived sAML, cohort C). There was a clear discrimination between the three categories regarding median survival (unpublished observation). These findings suggest that the three categories chosen in this study really represent different stages of CMML evolution. Finally, data in the biodatabase are from samples of patients who had sometimes repetitive sampling. Whereas this may cause some sampling bias in some analyses it also offers the possibility to monitor the course of disease in individual patients.

Despite all the limitations mentioned above there is general consensus now that real-world data should be increasingly used particularly in rare diseases to exploit this ever richer source of information [30]. Studies regarding treatment outcome in these patients are planned in the future. This study demonstrates that the cohort of CMML patients collected in the ABCMML shares clinically relevant characteristics with reported CMML cohorts from other countries. Therefore, results from molecular and biologic analyses using material from this national cohort of CMML patients will also be applicable to other CMML series and thus have a more general significance.
